# CRISPR-Cas9: Tool for Qualitative and Quantitative Plant Genome Editing

**DOI:** 10.3389/fpls.2016.01740

**Published:** 2016-11-21

**Authors:** Ali Noman, Muhammad Aqeel, Shuilin He

**Affiliations:** ^1^College of Crop Science, Fujian Agriculture and Forestry UniversityFuzhou, China; ^2^Department of Botany, University of AgricultureFaisalabad, Pakistan; ^3^National Education Minister Key Laboratory for Plant Genetic Improvement and Comprehensive Utilization, Fujian Agriculture and Forestry UniversityFuzhou, China

**Keywords:** CRISPR, plants, genome editing, targeted modifications, novel phenotypes

## Abstract

Recent developments in genome editing techniques have aroused substantial excitement among agricultural scientists. These techniques offer new opportunities for developing improved plant lines with addition of important traits or removal of undesirable traits. Increased adoption of genome editing has been geared by swiftly developing Clustered regularly interspaced short palindromic repeats (CRISPR). This is appearing as driving force for innovative utilization in diverse branches of plant biology. CRISPR-Cas9 mediated genome editing is being used for rapid, easy and efficient alteration of genes among diverse plant species. With approximate completion of conceptual work about CRISPR-Cas9, plant scientists are applying this genome editing tool for crop attributes enhancement. The capability of this system for performing targeted and efficient modifications in genome sequence as well as gene expression will certainly spur novel developments not only in model plants but in crop and ornamental plants as well. Additionally, due to non-involvement of foreign DNA, this technique may help alleviating regulatory issues associated with genetically modified plants. We expect that prevailing challenges in plant science like genomic region manipulation, crop specific vectors etc. will be addressed along with sustained growth of this genome editing tool. In this review, recent progress of CRISPR-Cas9 technology in plants has been summarized and discussed. We reviewed significance of CRISPR-Cas9 for specific and non-traditional aspects of plant life. It also covers strengths of this technique in comparison with other genome editing techniques, e.g., Zinc finger nucleases, Transcription activator-like effector nucleases and potential challenges in coming decades have been described.

## Introduction

Genome editing (GE) encompasses numerous techniques of immense value for plant genome modifications. These techniques enable us to change the gene expression regulation at pre-determined sites and facilitate new insights into the plant functional genomics. GE differs from genetic engineering. So, no foreign DNA is made part of plants and they cannot be distinguished from parent plants. Genome engineering of plant cell lines or plant models has conventionally been achieved either through random mutagenesis or low-efficiency gene targeting (Hsu et al., 2014; Ma et al., 2014; Sprink et al., 2015; Wolt et al., 2016). Genome editing includes a wide variety of tools. Making the genome editing practical and reliable, techniques like Genome editing with engineered nucleases (GEEN) and programmable sequence-specific DNA nuclease etc. have granted precision to process of endogenously targeted genomic modifications. The versatile genome-editing tool CRISPR (Clustered regularly interspaced short palindromic repeats) is a comparatively precise approach to modify DNA at specific sites. CRISPR has evolved as principal technique for gene function analysis and genesis of genetic variation (Deltcheva et al., 2011; Perez-Pinera et al., 2013; Kanchiswamy et al., 2016). Particularly, success in genome modification has been noticed among species that are difficult to be modified by other techniques (Bolotin et al., 2005; Xing et al., 2014). To date, most of the studies have been conducted by using animal systems. During last few years, CRISPR-Cas9 mediated mutagenesis was performed in *arabidopsis*, sorghum, tobacco, proving applicability of this technique to both dicot and monocot plants (Feng et al., 2013; Li et al., 2013). Generally, CRISPR-Cas9 is highly adaptable for editing of plant genome (Charpentier and Doudna, 2013; Schaeffer and Nakata, 2015) but especially appropriate for genome editing of monocotyledons, e.g., rice due to high genomic GC content (Miao et al., 2013). With special reference to economically valuable plants, i.e., crops and ornamentals, this technique offers an extraordinary and pragmatic system to produce novel phenotypes. CRISPR together with Cas proteins form the CRISPR-Cas system (Zhou et al., 2014; Bortesi and Fischer, 2015).

The functions of CRISPR and Cas genes (CRISPR-associated) are indispensable for adaptive immunity in some bacteria and archaea. These act as facilitator in response to viral genetic material. Discovered in 1980s in *Escherichia coli* (Ishino et al., 1987), function of these repeats was confirmed in 2007. Till now, workers across the world have described three types of mechanisms. Type II of CRISPR is the most studied type (Bortesi and Fischer, 2015). The Types I and III system involves specific Cas endonucleases which make the pre-crRNAs (Pre-CRISPR RNA) and after attaining maturity, this crRNA assembles into Cas protein complex. This complex possesses ability to recognize and cleave nucleic bases complementary to the crRNA (Jinek et al., 2012). The CRISPR-Cas9 type II is characterized as small RNA-based immune system of archaea and bacteria (Haft et al., 2005). CRISPR-Cas9 system is featured by relative construction simplicity along with high functional efficiency in human, animal, and plant cells (Nemudryi et al., 2014). The technique allows access target recognition by using gRNAs instead of synthetic DNA-binding domains. This characteristic makes it simple in comparison with ZFNs and TALENs (Cong et al., 2013; Wang et al., 2013).

Genome editing is being adopted for economically significant plants with full trust in terms of technical viability, dogmatic acceptance and profit-making practicability (Miao et al., 2013; Bortesi and Fischer, 2015). It is noteworthy that different genetic engineering techniques can leave behind DNA alteration traces. The crop plants and ornamentals generated by means of genome editing can escape the strict statutes and regulations generally associated with GM plant development. Due to this reason many researchers believe that improvements in plant varieties through precise genome editing techniques will be highly acceptable to the public as compared to transgenic plants (Abdallah et al., 2015).

The advent of CRISPR has made it possible to rewrite host DNA by introducing some major modifications. These modifications include gene replacement, deletions, inversion, knockouts, and translocations. But more prominent are the potential prospects of this technique for producing plants with mutations linked to other disciplines of science, i.e., synthetic biology, biofuel production, disease resistance, abiotic stress tolerance, phytoremediation etc. The establishment of plants with desired gene modifications can pave the way to study complex plant biology. Unfortunately, plant science is far behind than other disciplines in application of this technology. Therefore, keeping in view the immense importance of this technique, we have summarized the prospective role of CRISPR-Cas9 for plants and related benefits. A brief comparison of CRISPR-Cas9 and other genome editing techniques has been made to justify its strengths. We attempted to sum up current progress in CRISPR-Cas9 technology especially in plant biology and potential challenges for future development.

## Glimpses From History

**FIGURE 1:**
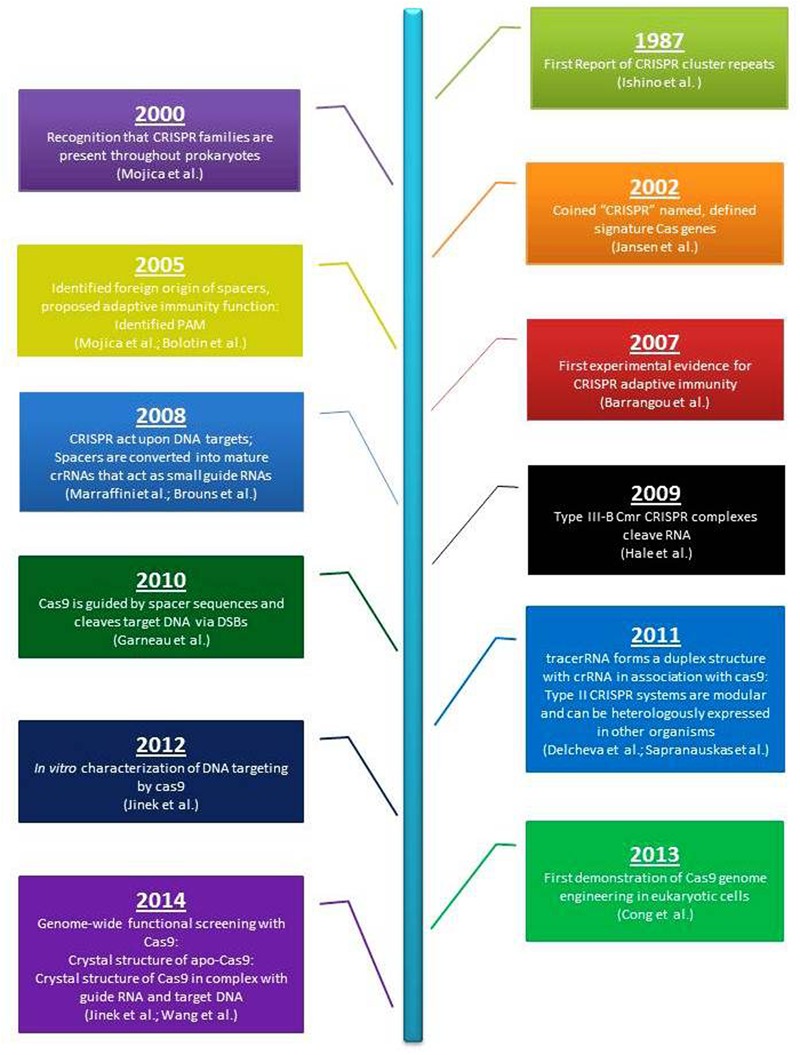
**Two decades of CRISPR-Cas9 adoption and success**.

High frequency of plant genome editing is evident in economically significant plant species due to practical feasibility and viability (**Figure 1**). Initially, evidences of earliest genome editing were expressed with oligonucleotide mediated mutagenesis (OMM) for herbicide resistance in rice, maize, tobacco etc. (Kochevenko and Willmitzer, 2003; Iida and Terada, 2005; Sander and Joung, 2014; Wolt et al., 2016). Gao et al. (2010) used engineered mega nuclease (EMN) for editing maize genome by using native endonuclease altered to identify and tempt very specific DSBs (Double stranded breaks) at definite locus. This resulted in disruption of gene in terms of indels (Insertions-deletions) by non-homologous end joining (NHEJ).

In addition to this, successful target gene insertion for delivery of herbicide tolerance in cotton had been carried out through yeast endonuclease engineered EMN (D’Halluin et al., 2013). Afterward, it was observed that ZFN based site-specific trait stacking strategy produced excellent results in maize in form of new plant line possessing herbicide resistant gene.

Finally, accomplishment of CRISPR-Cas9 technique was observed for competent targeted mutagenesis in transgenic rice for improvement in growth and yield. Researchers have successfully demonstrated the production of transgenic rice having mutations in particular genes by adopting CRISPR-Cas9 technology (Miao et al., 2013; Zhang et al., 2014; Wolt et al., 2016). Xing et al. (2014) presented a toolkit for facilitating transient/stable expression of CRISPR-Cas9 in diverse plant species. The year 2016 mark the finalization of herbicide tolerant canola, e.g., Cibus 5715 approved for cultivation in Canada.

Now, several research groups have focused application of CRISPR technology on plants of significant economic worth such as rose, apple, potato, egg plant, rice (**Table 1**) (Wendt et al., 2013; Char et al., 2015; Sprink et al., 2015; Xiong et al., 2015; Kanchiswamy et al., 2016). Unequivocally, this technique is efficient, well-organized and flexible for editing multiplex gene. Now time is to focus on application of CRISPR-Cas9 system to other cereals with larger and complex genomes, e.g., wheat, sugar cane. Parallel with this, improvements in this technique, i.e., elimination of CRISPR-Cas9 remains after target genes mutation, will support the usage of this tools in agriculture.

**Table 1:** Successful application of CRISPR-Cas9 in different plant species.

DNA modification type	Plant	Delivery mode	Target(s)	Gene function(s)	Reference
Gene Knockout: rewriting of host DNA	*A. thaliana*	Stable integration	*RTEL1*(Regulator Of Telomere Elongation Helicase 1)	***RTEL1*** functions in DNA replication, DNA repair, and recombination	Schiml et al., 2014
	*A. thaliana*	Stable integration	*AP1* (floral homeotic *gene* APETALA1), *BRI1* (Brassinosteroid-insensitive2), *CHLI1*(Magnesium-chelatase subunit ChlI-1)	***BRI1*** encodes a cell surface receptor for brassinosteroids.***AP1*** Encodes a putative TF that acts locally to specify the identity of the floral meristem. ***CHLI1*** plays role in chlorophyll biosynthesis.	Feng et al., 2014
	*A. thaliana*	Stable integration	*ADH1* (Alcohol dehydrogenase class-P)	***ADH1*** is required for survival and acclimation in hypoxic conditions, especially in roots	Fauser et al., 2014
	*A. thaliana N. benthamiana*	Protoplasts, Agrobacterium T-DNA(Transient)	At *Phytoene desaturase* gene (PDS*3*), *NbPDS3*	***PDS3*** is needed for primary carbon and pigment metabolism. Its activity acts as a rheostat of retrograde signaling during early chloroplast biosynthesis.	Li et al., 2013
	*O. sativa, S. bicolor*,	Protoplasts, Agrobacterium T-DNA (Transient)	*OsSWEET14*,	***OsSWEET14*** id needed for disease resistance.	Jiang et al., 2013
	*O. sativa T. aestivum*	Protoplasts	*OsPDS,OsBADH2* (betaine aldehyde dehydrogenase-2), *OsMPK2* (ortholog of tobacco SIPK), *TaMLO* (Mildew-resistance locus)	***BADH2*** *plays important role in abiotic stress tolerance.* ***OsMPK2*** is activated by elicitors *It* negatively regulates the expression of defense-related genes. ***TaMLO in*** plant confers heritable broad-spectrum resistance to powdery mildew	Shan et al., 2013c
	*A. thaliana, O. sativa*	Stable integration	*AtJAZ1* (Jasmonate ZIM-domain), *(Gibberellic Acid insensitive),OsROC5* (RICE OUTMOST CELL-SPECIFIC GENE 5), *OsSPP, OsYSA*	*AtJAZ1 is* transcription repressor of jaJA)-responsive genes and major components of theJA receptor complex. ***AtGAI*** *is important in* attenuation of GA responses.	Feng et al., 2013
	*O. sativa*	Stable integration	*OsWEET11/13/1a/1b*	***OsSWEET14*** id needed for disease resistance.	Zhou et al., 2014
Large deletions:	*A. thaliana*	Protoplasts	*PDS3*	***PDS3*** is needed for primary carbon and pigment metabolism.	Li et al., 2013
Gene replacement:	*O. sativa*	Protoplasts	*Phytoene desaturase* gene	***PDS3*** is needed for primary carbon and pigment metabolism.	Shan et al., 2013a
	*N. benthamiana*	Protoplasts	*Phytoene desaturase* gene	***PDS3*** is needed for primary carbon and pigment metabolism.	Li et al., 2013
	*A. thaliana*	Stable integration	*ADH1 (Alcohol dehdrogenase1)*	***ADH1*** is required for survival and acclimation in hypoxic conditions, especially in roots	Schiml et al., 2014
Controlling gene expression	*N. benthamiana*	Agrobacterium T-DNA (Transient)	*PDS3*	***PDS3*** is needed for primary carbon and pigment metabolism. Its activity acts as a rheostat of retrograde signaling during early chloroplast biosynthesis.	Piatek et al., 2014

## Mechanism of CRISPR-Cas9 Based Genome Editing

CRISPR-Cas9 system just requires three components, i.e., Cas9, tracer RNA (trRNA), CRISPR RNA (crRNA) for function. This prospective was recognized in start of this decade (Jinek et al., 2012; Hsu et al., 2014; Schaeffer and Nakata, 2015). In type II of CRISPR, attacking viral DNA or plasmids is divided into smaller pieces and integrated in CRISPR locus. The particular loci are transcribed and processed transcripts produce crRNA. These crRNAs direct effector endonuclease to target alien DNA depending upon complementarity of sequence. Cas9 produce DSBs (double-stranded breaks) at target site, which on the other hand facilitates endogenous DNA repair mechanisms leading to edited DNA (Charpentier and Doudna, 2013; Hsu et al., 2014).

**FIGURE 2:**
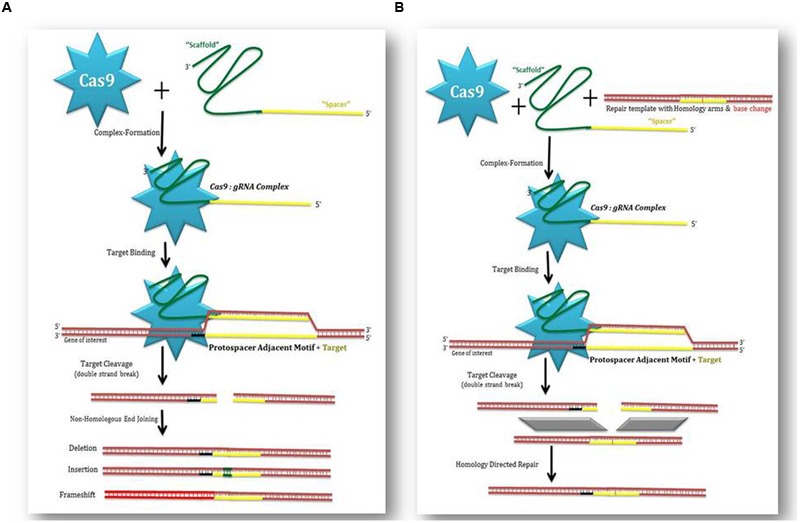
**How CRISPR-Cas9 perform genome editing.** Cas9 induce double stranded breaks (DSBs) at particular site. The resulting DSB is then repaired by one of these two general repair pathways, e.g., by Non-homologous end joining (NHEJ) or by Homology directed repair (HDR). **(A)** The NHEJ repair pathway frequently results in small nucleotide insertions or deletions (InDels) at the DSB site. This may result in gene knock out or gene insertion. **(B)** HDR can be used to generate precise nucleotide modifications (also called gene “edits”) ranging from a single nucleotide change to large insertions.

Type II system, comprises of crRNA and trRNA that combine into one sgRNA (single guide RNA) (Jinek et al., 2012; Xing et al., 2014). Amazingly, the sgRNA programmed Cas9 appeared more effective in targeted gene modifications rather than individual trRNA and crRNA. Till today, genome-editing protocols have adopted three different types of Cas9 nuclease. The first Cas9 type can cut DNA site-specifically and results in the activation of DSB repair. Cellular NHEJ (Non-Homologous End Joining) mechanism is used to repair DSBs (Hsu et al., 2014; Sternberg et al., 2014). As a consequence, insertions/deletions (indels) takes place that interrupt the targeted loci (**Figure 2**). Otherwise, if any similarity between donor template and target locus is witnessed, the DSB may be mended by HDR pathway (homology directed repair) allowing exact substitute mutations to be prepared (Hale et al., 2009; Sternberg et al., 2014; Schaeffer and Nakata, 2015). Cong et al. (2013) introduced advanced Cas9-D10A, a mutant form having more précised nickase activity. It cuts single strand of DNA without activation of NHEJ. As an alternative, DNA repairs took place via the HDR pathway only. Hence it produces less indel mutations (Jinek et al., 2012; Cong et al., 2013). Cas9-D10A is very target specific particularly when any locus is encountered by paired Cas9 complexes for generation of contiguous DNA nicks (Ran et al., 2013). The third type is dCas9, nuclease-deficient Cas9 (Qi et al., 2013). Although mutations in the HNH domain and RuvC domain discharge cleavage activity, but do not prevent DNA binding (Gasiunas et al., 2012). Therefore, this particular variant can be utilized in sequence-specific targeting of any genome regardless of cleavage. In its place, dCas9 may be taken as a tool for either gene silencing or activation by fusion with a variety of effector domains (Maeder et al., 2013a,b). One bonus of this technique is the case of not using recombinant DNA (**Figure 2**). This situation can result in edited plants exempted from current GMO regulations. So we can hope for widespread application of RNA-guided genome editing in agriculture and plant biotechnology.

## Why CRISPR-Cas9 is More Trustworthy than Talens and ZFNs?

For assessment of any genome editing tool, % age of achieved desired mutation known as Targeting efficiency (TE) is regarded as the most reliable attribute. The success ratio of Cas9 TE can be compared with other techniques like TALENs or ZFNs (Wendt et al., 2013; Ma et al., 2014). For example in human cells, custom-designed ZFNs and TALENs could only achieve 1–50% efficiencies (Maeder et al., 2008; Miller et al., 2011; Wolt et al., 2016). Conversely, TE of Cas9 in animals and plants, respectively, i.e., zebrafish, maize has been observed up to 70% and it ranges between 2 and 5% in case of induced pluripotent stem cells (Fu et al., 2013; Hsu et al., 2013). Later on,CRISPR-Cas9 efficiency was recorded up to 9.2% as compared to ZFN efficiency that was lower than 1% in case of pigs IGF2 (Insulin-like growth factor 2). Reports are available that broadly describe better genome targeting of single cell mouse embryo up to 78% and successful effectual germline transmission by using dual sgRNAs (Zhang et al., 2013; Zhou et al., 2014; Wolt et al., 2016). Moreover, incidence of off-target mutations is also an effective parameter for assessment of genome editor’s performance. Such mutations may be observed in sites that have dissimilarity of small number of nucleotides in comparison with original sequence till they are neighbors of Protospacer adjacent motif (PAM) sequence. The DNA sequences are used to transcribe crRNA targeting sequences known as protospacers. These consist of short sequences and found clustered in bacterial genome in form of a group called CRISPR array. The PAM sequence is absolute need of Cas9 for binding its target. Cas9 do not cleave the protospacer sequence in absence of adjacent PAM sequence. This favors the stance that Cas9 can endure mismatches up to five bases within the protospacer region (Fu et al., 2014; Sander and Joung, 2014) or one base divergence in the PAM sequence (Hsu et al., 2013). Other than facilitation activity for genome alterations, the wild-type Cas9 nuclease has capacity to be transformed into dCas9 after inactivation of catalytic domains. Furthermore, effector fusion usage can enhance the range of genome engineering modalities attainable by adopting Cas9. Normally, off-target mutations are bit difficult to detect because these require full genome sequencing to completely rule them out. So unanimous opinion is CRISPR-Cas9 facilitates plant genomes interrogation, as it enables high efficiency generation of mutants bearing multiple gene mutations (**Tables 2** and **3**). This effective approach endorses high specificity of wide range genome editing applications.

**Table 2:** Tabular presentation of comparative attributes of plant genome editing techniques.

	CRISPR/Cas9	Zinc Finger Nucleases (ZFNs)	Transcription factor like effector nucleases (TALENs)	Reference
Mode of action	It works by inducing double-strand breaks in target DNA or single-strand DNA nicks (Cas9 nickase).	It can induce double-strand breaks in target DNA.	Induces DSBs in target DNA.	Li et al., 2013; Mao et al., 2013
Off target effects	These effects can be minimized by selecting unique crRNA sequence.	These have off-target effects.	Off target effects cannot be avoided.	Hsu et al., 2013
Generation of large scale libraries	YES, this is possible to generate large scale libraries.	Such generation is not possible because it requires customization of protein component for each gene.	Generation of large scale libraries is possible but technically difficult and challenging.	Cho et al., 2013; Hsu et al., 2013
Protein engineering steps	It does not requires protein engineering steps, very simple to test multiple gRNA.	It requires complex to test gRNA.	TALENs need protein engineering steps to test gRNA.	Cho et al., 2013
Cloning	Cloning is not necessary.	Cloning is necessary.	It requires cloning.	Cho et al., 2013
gRNA production	Any number of gRNA can be produced by *in vitro* transcription. It keeps budget away from extra load.	Bit difficult to produce this kind of RNA.	gRNA production is bit difficult to achieve through these effector nucleases.	Cho et al., 2013
Methylated DNA cleavage	It can cleave methylated DNA in human cells. This aspect is of special concern for plants as this has not been much explored	Unable to do so.	There are many question marks upon capacity of TALENs to perform methylated DNA cleavage.	Hsu et al., 2013; Ding et al., 2013
Multiplexing	This is main advantage of CRISPR. Several genes can be edited at same time. Only *Cas9* needed	Highly difficult to achieve this through *ZFNs*.	Very difficult to obtain multiplexed genes by means of TALENs. Because it needs separate dimeric proteins specific for each target	Li et al., 2013; Mao et al., 2013
Structural proteins	*CRISP R* consists of single monomeric protein and chimeric RNA.	*ZFNs* work as dimeric and only protein component required.	TALENs *also* work as dimeric and require protein component.	Li et al., 2013; Upadhyay et al., 2013; Zhou et al., 2014
Catalytic domain	It has two cleavage domains called RUVC and HNH.	*ZFNs* have catalytic domain of restriction endonuclease FOKI which generates a DSB.	TALENs also have FOKI catalytic domain of restriction endonuclease for DSB generation.	Jinek et al., 2012
Mutation rate	Comparatively low mutation rate has been observed.	High mutation rate observed in plants.	Mutation rate is high as compared to CRISPR.	Li et al., 2013
Components	crRNA, Cas9 proteins	Zn-finger domains Non-specific FokI nuclease domain	Zn-finger domains Non-specific FokI nuclease domain	Kumar and Jain, 2014
Length of target sequence (bp)	20–22	18–24	24–59	Chen et al., 2016
Target recognition efficiency	High	High	High	Kumar and Jain, 2014
Level of experiment setup	Easy and very fast procedure of designing for new target site	Complicated procedure of redesigning for each new target site and need for expertise in protein engineering	Relatively easy procedure of designing for each new target site	Kumar and Jain, 2014

**Table 3:** Technical limitations in CRISPR-Cas9 application and their effects.

Pitfall	Reason (s)	Effects	Reference
Off-target effects	**(1)** Improper concentration ratio between Cas9 and sgRNA may cause off-target cleavage. **(2)** PAM sites may lead to undesired cleavage of DNA regions.	Unexpected mutations	Sternberg et al., 2014
Cas9 codons	Insufficient Cas9 codon optimization	Inefficient translation of Cas9 proteins	Feng et al., 2013, 2014; Schiml et al., 2014
Vectors	Mostly CRISPR/Cas9 systems use exogenous promoters for Cas9 and sgRNA expression. Vectors with optimal promoters should be selected.	Improper vectors can stop system proceedings.	Shan et al., 2014
Gene homologs	Gene family members may complicate target sequences to be edited.	False editing of target sequence.	Song et al., 2016
Epigenetic factors	DNA methylation or histone modification occurs not in regions with complex DNA compositions, such as those with repetitive sequences.	limit protein binding or RNA pairing	Song et al., 2016

## Applications of CRISPR-Cas9 in Plant Biology and Biotechnology

The application of CRISPR-Cas9 has made it possible to rewrite host DNA by introducing some major alterations in plant genomes. Use of CRISPR-Cas9 is facilitating multiple ranges of genome engineering applications (**Tables 4** and **5**). Plant species with intractable genomes have now been targeted with Cas9 nuclease for introduction of various levels of genome modifications. Here, we will take into account prospective role of CRISPR-Cas9 in editing plant genome for achieving broad range goals.

**Table 4:** List of promoters and gene(s) targeted through CRISPR-Cas9 system in different plants.

Plant	sgRNA Promoter(s)	Cas9 Promoter(s)	Target (s)	Reference
*Triticum aestivum*	TaU6	2 × 35S	*TaMLO* (Wheat Mildew-resistance locus)	Shan et al., 2013b
	TaU6	Ub1	*TaMLO-A1* (Wheat Mildew-resistance locus1)	Wang et al., 2014
*Citrus sinensis*	CaMV 35S	CaMV 35S	*CsPDS* (*Phytoene desaturase* gene)	Jiang and Wang, 2014
*Sorghum bicolor*	OsU6	Rice Actin1	*DsRED2* (Red fluorescent protein)	Jiang et al., 2013
*Nicotiana benthamiana*	AtU6	35DPPDK	*NbPDS3* (Tobacco *Phytoene desaturase* gene)	Li et al., 2013
	OsU6	35S	*GFP* (Green fluorescent Protein*)*	Jiang et al., 2013
	CaMVE35S	CaMVE 35S	*Nb* PDS3 (*Phytoene desaturase* gene)	Upadhyay et al., 2013
*Marchantia polymorpha* L.	MpU6-1	CaMV 35s and MpEF1α	*MpARF1*	Sugano et al., 2014
*Arabidopsis thaliana*	AtU6	35DPPDK	*AtPDS3, AtRACK1b* (Receptor for activated C kinase 1) *andAtRACK1c* (Receptor for activated C kinase 1c)	Li et al., 2013
	AtU6-26	2 × 35S	*BRI1* (Brassinosteroid-insensitive2), *JAZ1* (Jasmonate ZIM-domain), *and YFP*	Feng et al., 2013
	OsU6	35S	*GFP* (Green fluorescent Protein)	Jiang et al., 2013
*Oryza sativa*	OsU3	2 × 35S	*OsPDS3*,Os*BADH*2 (betaine aldehyde dehydrogenase-2), *Os02g23823 and OsMPK2* (ortholog of tobacco SIPK)	Shan et al., 2013c
	OsU6	CaMV 35S	*OsSWEET11 and OsSWEET14* (Rice bacterial blight susceptibility genes)	Jiang et al., 2013
	OsU3	Ub1	*CAO1 and LAZY1*	Miao et al., 2013
	OsU6-2	35S	*ROC5* (Rice outmost cell-specific gene 5), *SPP and YSA*	Feng et al., 2013

**Table 5:** Different plasmids with their genes, vectors, and promoters used in CRISPR-Cas9 technique.

Plasmids	Gene/Insert	Promoter	Vector type	Vector back bone	Purpose	Bacterial resistance	Reference
pK7WGF2::hCas9	hCas9 (Syn)	35S	Plant expression, CRISPR	pK7WGF2,	Expresses the human codon by using Cas9 with N-terminal GFP tag from the 35S promoter in the plant tissue	Spectinomycin	Nekrasov et al., 2013
PHSE401	zCas9, gRNA scaffold	35S, AtU6-26p	Plant Expression; plant binary vector	pCambia,	CRISPR/Cas based plant genome editing and gene regulation	Kanamycin	Xing et al., 2014
pHEE401E	zCas9, gRNA scaffold	U6-26p *Arabidopsis* U6 gene promoter, EC1.2 enhancer fused to EC1.1 promoter	Plant Expression; plant binary vector, CRISPR	pCambia	Contain gRNA scaffold for insertion of target sequence, Egg cell-specific promoter-controlled expression of 3×FLAG-NLS-zCas9-NLS	Kanamycin	Wang et al., 2015
pHSN501	zCas9D10A, gRNA scaffold (Syn)	AtU6-26p, 2 × 35Sp	CRISPR; Plant expression	pGreen-like binary vector	CRISPR/Cas based plant genome editing and gene regulation; expresses zCas9D10A	Kanamycin and Spectinomycin	Xing et al., 2014
pBUN501	gRNA scaffold (Syn), zCas9D10A (Syn)	AtU6-26p, Ubi1p,	Plant expression, CRISPR	pGreen-like binary vector	CRISPR/Cas based plant genome editing and gene regulation;	Kanamycin and Spectinomycin	Xing et al., 2014
pHSN6A01	gRNA scaffold (Syn),dCas9-VP64 (Syn)	AtU6-26p, 2 × 35Sp,	Plant expression, CRISPR	pGreen-like binary vector	expresses dCas9-VP64, gRNA scaffold for insertion of target sequence	Kanamycin and Spectinomycin	Xing et al., 2014
pBUN6A11	gRNA scaffold (Syn), dCas9-VP64 (Syn),	OsU3p, Ubi1p,	Plant expression, CRISPR	pGreen-like binary vector	expresses dCas9-VP64, gRNA scaffold for insertion of target sequence	Kanamycin and Spectinomycin	Xing et al., 2014
pEGB 35s:dCas:BRD:tNos (GB1172)	dCas9:BRD	35S	Plant expression, CRISPR	pDGB3alpha2	Transcriptional unit of (human codon optimized) inactivated Cas9 fused to the BRD Transcriptional Repressor	Kanamycin	Vazquez-Vilar et al., 2016
pEGB 35S:dCas9:Tnos (GB1191)	dCas9	35S	Plant Expression, CRISPR, Synthetic Biology	pDGB3alpha2	Transcriptional unit for human codon optimized with mutated (D10A, H840A) and inactivated catalytic domains Cas9 protein plant expression driven by the 35S promoter	Kanamycin	Sarrion-Perdigones et al., 2013
pJIT163-2NLSCas9	dCas9	2 × 35S	Plant Expression	pJIT163	Expression of rice codon-optimized Cas9 in plant cells	Ampicillin	Shan et al., 2013b
HBT-pcoCas9	Pro Cas9 (syn)	Hybrid constitutive promoter 35SPPDK	CRISPR; Plant expression	HBT-FLAG	Transient expression of pcoCas9 gene in plant cells	Ampicillin	Li et al., 2013

### Crispr-Cas9 and Plant Synthetic Biology

Ranging from production of primary metabolites necessary as food to secondary metabolites, plant based products are of great concern for multiple purposes. With ongoing progress in the field of plant biology in general and synthetic biology particularly, researchers are seeking to produce novel biological systems, inclusive of industrially designed plant cells and plants. One of the chief targets sets for synthetic biology is the wish for minimal plant cell, e.g., to engineer a cell devoid of non-essential components and capable of division. This desired minimal cell can then be exploited as a factory for novel biological systems. Although this minimal cell is still a dream, the prospective toolkits and strategies for generating the simplest plant cell are being operated recurrently (Baltes et al., 2015). Up till now, genome editing had been restricted to amendments in enzymatic functions within single animal or plant. Synthetic biology has already been using bacteria for engineering new absolute metabolic cycles comprising of both several enzymes and regulation of corresponding genes expression. CRISPR-Cas9 provide the most reliable and practical platform to engineer plant genome for multipurpose plant systems (Puchta and Fauser, 2014). Nitrogen fixing cereals project is classical example of goal fixed for humanity level benefits. The possibilities of genetic and metabolic engineering have been extended as a result of techniques developed for facilitating synthetic biology, especially cloning and genome editing methods (Neumann and Neumann-staubitz, 2010). At John Innes centre, different pathways have been characterized for plants capable of fixing nitrogen through bacteria (Oldroyd et al., 2011; Xie et al., 2012). The researchers are now attempting to introduce these pathways in wheat for developing ‘self-fertilizing’ cereal (Cook et al., 2014). By doing so, there will be clear cut reduction in dependency upon inorganic fertilizers because plants will be able to fix atmospheric nitrogen. At this time, there are two possible ways: either transfer the Nod factor signaling pathway to cereals or relocate the nitrogenase enzyme from nitrogen fixing bacteria into plant cells. But still different questions need to be addressed (Temme et al., 2012; Oldroyd and Dixon, 2014). A potential goal set by plant synthetic biology is C4 rice development with the help of targeted DNA insertion. Engineering rice with C4 photosynthesis pathway appears promising for increasing yield. One line of action to engineer this pathway in C3 rice is conversion of single-cell C3 cycle into a two-celled C4 cycle. The initial carbon fixation is carried out within mesophyll cells. Finally the four-carbon product is decarboxylatedforCO_2_ addition to RuBisCO present in bundle sheath cells. (Baltes et al., 2014). CRISPR-Cas9 has been more successfully applied to mutagenize host DNA in different plants (Li et al., 2013; Jiang et al., 2014; Zhou et al., 2014). The ability to introduce genomic amendments encourage synthetic biologists not merely remove unwanted DNA, i.e., inhibitory genes but also improve genic regulatory sequences.

Practicing plant synthetic biology needs control over nucleotide sequences in plant as well as control over expression levels of host genes. The DNA binding domain of different sequence- specific nucleases can be repurposed to help in modulation of endogenous genes expression. DNA-binding domains from ZFNs, TALENs, or dCas9 and gRNA are used to limitize repressor or activator domains in gene of interest. Uniquely, Cas9 by interfering with RNA polymerase progression can decrease gene expression (Qi et al., 2013).

The site-specific integration of DNA into plant genomes will be of special significance for plant synthetic biology research that demands the transfer of a number of genetic segments for conferring new biological function (Baltes and Voytas, 2015). CRISPR-Cas9 cannot only make traits stacking easy but also reduce variability in gene expression. Due to least targeting limitations for CRISPR-Cas9 system, almost all chromosomal positions are amenable to site-specific integration (Baltes and Voytas, 2015). First of all, transgene stacking was demonstrated in maize. After co-transformation of immature maize embryos with donor DNA and DNA encoding the ZFNs, 5% of transgenic progeny witnessed proper integration (Ainley et al., 2013). Similarly, trait stacking has successfully been done in cotton (D’Halluin et al., 2013). Other than integrating genes with the help of homologous recombination, NHEJ can be used for targeted gene insertion (Baltes et al., 2014). But this approach has not been extensively applied in plants.

However, several challenges are still to be addressed. Most importantly, successful plant system engineering and development will depend on suitable and efficient delivery systems by targeting specific tissues. There is need is to develop techniques providing command over the triplet code, therefore likely to enable us for selected amendments in DNA sequence within plant cells. We are convinced that editing genome is going to exert a material influence on the valuable scheme for plant trait improvement. It will be of supreme significance to systematically characterize the safety as well as physiological effects of Cas9 in plant synthetic biology by adopting a variety of methods.

### Crispr-Cas9, a Perspective Strategy for Plant Genome Imaging

Visible genome imaging is largely executed to measure features of plant genome architecture (Sozzani et al., 2010). The intracellular organization of structural and functional elements contributes to the genomic functional output which can be dynamically enhanced or concealed. The physical genome organization has appeared specifically significant but still reckoned as obscure mechanism. Researchers used different methods like chromosome conformation capture (3C) to answer various genome related queries. 3C derived techniques, e.g., Hi-C have introduced innovative insights into genome spatial organization principles inclusive of the presence of TADs (topologically associated domains) (Chen et al., 2016). Genomic loci positioned mega bases away on same or different chromosomes could be brought closer given apt chromosomal organization, consequently mediating lengthy *trans* interactions (Ma et al., 2014). Conversely, there is still question mark upon manner for genome modification and *in vivo* modulation of their structural organization afterward (Malina et al., 2014). But, without vital methodology for DNA visualization, studying various gene interactions in different chromatin states would merely be a dream. CRISPR type II resulting from *Streptococcus* (Wiedenheft et al., 2012) is capable of achieving this goal (Chen et al., 2016). Griffith et al. (1999) conducted series of experiments for verification of telomere imaging efficiency and specificity by CRISPR technique. The number of telomeres identified with the help of CRISPR or specific Fluorescence *in situ* hybridization (FISH) with peptide nucleic acid (PNA) was similar. Thus, the matched score for cell imaging indicated similar efficiencies of FISH and CRISPR for cellular imaging. This finding declares CRISPR as an optimized toolkit for telomere visualization along with role in gene regulation enhancement. Traditional DNA labeling techniques like FISH require sample fixation and therefore, incapable to capture live course of actions. Labeling of particular DNA loci with the help of fluorescently tagged Cas9 had been introduced as a potent live-cell-imaging substitute of DNA-FISH. dCas9-EGFP and sequence-specific sgRNAs co-expression facilitate the enhancement of fluorescent signals for imaging at targeted genomic loci (Chen et al., 2013, 2016; Hsu et al., 2014). CRISPR technique endow us with vigorous repetitive element between protein-coding genes, e.g., mucin genes and telomeres. Similarly, human genome non-repetitive elements have already been visualized with the aid of multiple sgRNA (Chen et al., 2013). Not alone but together with FISH or DNA-binding proteins, this CRISPR technique recommends a matching advancement for imaging. The capacity of CRISPR to tag human cell telomeres encouraged researchers to examine whether this technique can be used for assessing telomere length. A linear correlation was noticed between PNA based FISH and CRISPR for telomere length evaluation. The superiority of CRISPR over FISH is its ability to label telomere in addition to length measurement. This has been proved by correlation between telomere count and intensity (Chen et al., 2013).

**FIGURE 3:**
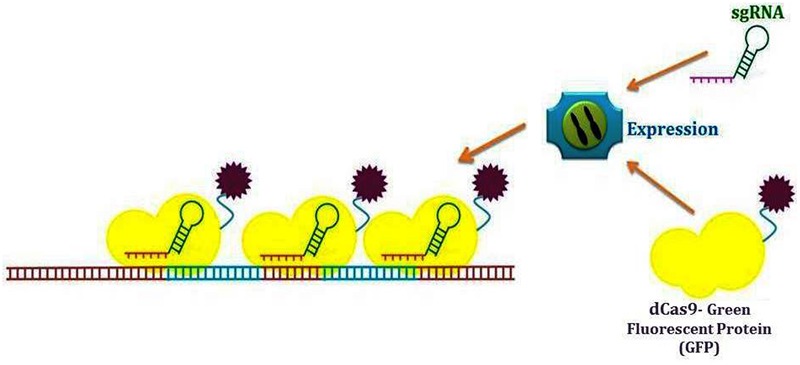
**Diagrammatic illustration of live-cell DNA labeling by using CRISPR-Cas9 system**.

Genome functional organization mapping can be greatly aided by techniques that are helpful in directly visualizing the interactions between various genomic elements, i.e., promoters or enhancers in living cells. Therefore, multicolor imaging method would be essential for imaging and tracking numerous genomic loci (**Figure 3**) (Chen et al., 2016). At present, two strategies have been devised for live cell imaging by using CRISPR-Cas9. The first strategy uses fluorescent Cas9 orthologs obtained from different bacterial types. The second strategy is by means of fluorescent RNA-binding protein joined to the sgRNA. Consequently, scaffold RNA (scRNA) is formed that encodes information about the target locus and the fluorescent color (Shao et al., 2016). These approaches have been successfully used for genomic regulatory programming (Konermann et al., 2015; Zalatan et al., 2015).

For genome imaging, RNA-guided Cas9 system specificity can be modified by replacing a small synthetic RNA without changing the protein component. Hence, designing and production of the labeling constructs become easy and very cost-effective (Chen et al., 2016). In comparison with TALENs, CRISPR-Cas system is more flexible for target site selection. A CRISPR-Cas9 target immediately precede PAM and usually starts with G (Jinek et al., 2012), while TALENs require T at the 5 end of the target sequence (Mak et al., 2012). For co-labeling of several loci, CRISPR-Cas9 is thus an easier choice.

Another advantage of CRISPR-Cas imaging is direct measurement of spatial information by loci positions imaging (Kalhor et al., 2012; Nagano et al., 2013). Therefore, it opens up new possibilities in studying chromosome conformation. CRISPR imaging can truly state a specific chromosome number by tagging a unique sequence to that specific chromosome and can precisely detect chromosome aneuploidy and mis-segregation (Chen et al., 2016). By using CRISPR imaging system and lineage tracking, we might check the aneuploidy growth kinetics with high temporal resolution in a population of given type of cells. Blend of dCas9 variants and diverse fluorescent proteins concur to label manifold genomic sequences within single genome (Chen et al., 2013). Hence, it enables us to obtain multicolor pictures for multiplexed finding of genetic actions. Prospective engineering of CRISPR can also make possible the plant RNAs recognition other than plant genomic DNAs. Additionally, chromosome translocation and transposition can be targeted for imaging with CRISPR (Roukos et al., 2013). Imaging with the help of CRISPR offer influential strategy to comprehend the heterochromatin formation control (Grewal and Jia, 2007).

CRISPR cas9 as an emerging technique of chromatin imaging present the aim to end the gap between sequencing studies and imaging studies. Even though technical challenges lie ahead, the prospective of CRISPR imaging will assist in solving many plant genome and chromatin related mysteries through direct cell imaging. CRISPR imaging’s unparalleled flexibility and accuracy in sequence targets lead us to accept as true the best is yet to come.

### Plant Epigenetic Responses and Crispr-Cas9

The dynamic events in epigenetic process determine multifaceted genome functions. Demand for dissection of plant multiple gene mutation is increasing. However, the existing methods for generating plants harboring several mutated genes involve time consumption and laborious efforts for genetic crossing of solitary-mutant plants (Xing et al., 2014; Quetier, 2015). Epigenetic alterations in DNA or histones that help in organizing chromosomes are expected to play vital roles in biological processes. Analysis of these modifications reveals their decisive value for transcriptional regulation and biological functions (Thakore et al., 2016). For example, epigenetic marks like acetylation and methylation at particular loci or histone residue can strongly effect gene expression. Responses such as histone acetylation and DNA methylation, are catalyzed by diversity of enzymes that are product of special genomic loci (Hsu et al., 2014; Song et al., 2016; Thakore et al., 2016). A multitude of enzymes can erase or produce epigenetic mark(s) on DNA. Few years back, zinc finger proteins and TAL effectors got attention and employed in many studies concerning with locus-oriented targeting of epigenetic amending enzymes (Maeder et al., 2013b; Mendenhall et al., 2013). Histone acetylation, as used in humans for transporting enzymes to specific place within genome, is of immense value and can also be used in plant epigenetics. Such epigenetic marks have particular effects (Ladford, 2016). The enzymes responsible for regulation of epigenetic state can be focused with the help of CRISPR based genome editing or used to produce genome wide perturbations in epigenetic state. This has already been observed in human embryonic stem cells (ESCs) after CRISPR-mediated observations of all DNA methyl transferases. This allows other researchers to characterize possible pluripotent cell lines with distinctive effects on the DNA methylation.

**FIGURE 4:**
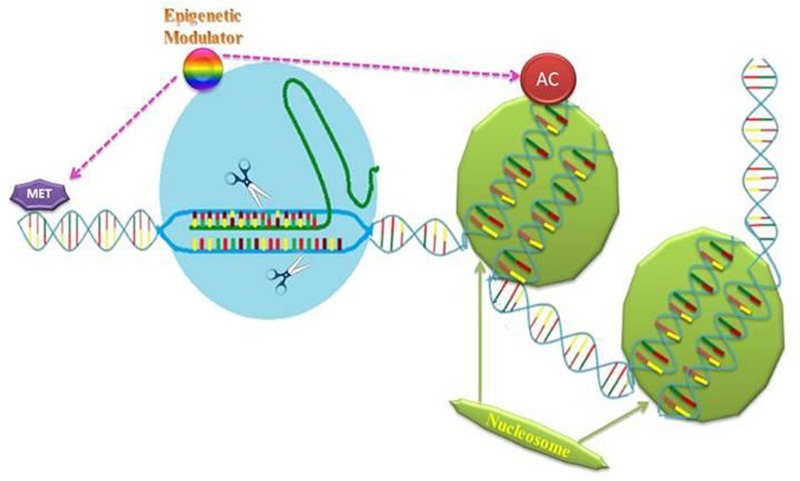
**Cas-9 has ability to be coupled with epigenetic modulators, i.e., that add acetyl group (Ac) to histones or that can add methyl group (Me) to DNA.** This will help researchers to find out role of precisely place modifications in effecting DNA dynamics or gene expression.

Researchers progressively need supplementary strategies for introducing epigenetic changes specifically at desired loci to test different hypotheses regarding potential implications of CRISPR-Cas technique in plant science. Epigenetic effectors are well able to cause covalent alterations to DNA and histones also (**Figure 4**). These can also turn on gene expression. Engineered ZFN and TALEN dependent thymine-DNA glycosylase (TDG) or 10-11 Translocation (TET) dioxygenases fusions may result in CpGs demethylation at target promoters. By this way, targeted DNA demethylation is induced, which facilitates re-activation target genes expression (Gregory et al., 2013; Chen et al., 2015; Li et al., 2015). The first observed targetable histone acetyl transferases were DBD–p300 core fusions. It is suggested that dCas9, TALENs and ZFNs fusion to p300catalytic core of histone acetyltransferase can activate gene expression from promoters as well as enhancers after depositing H3K27ac. Researchers are of the view that above mentioned fusion is predominantly important for transcriptional activation because multiplexing is not required. Additionally, distal enhancers can be activated that are unresponsive to dCas9-VP64 (Hilton et al., 2015; Thakore et al., 2016). H3K27ac augment gene expression aided by increased employment of activators in transcription along with transition of RNA Pol II to elongation from initiation. Thus enables achievement of transcriptional activation from targeted genes (Stasevich et al., 2014). Just like the affinity of the SAM complex for activation of transcription, epiCas9s (Cas9 epigenetic effectors) can also be applied for genome-wide screening to find out novel associations between chromatin states, DNA methylation and phenotypes, i.e., cellular differentiation (Hsu et al., 2014).

Definite epigenetic changes are adequate for influencing development of normal cells and play roles in later stages of plant development. Therefore, the enzymes for regulation of epigenetic alterations to histones or DNA can be special targets for normal plant development. CRISPR-Cas9 technology permits a catalytically inactive Cas9 to serve as targeted DNA-binding domain. When fused to epigenetic enzymes such as histone acetyl transferases (HATs) DNA methylases, or deacetylases (HDACs), the complex can simultaneously change the epigenetic state in a accurate way at a single or several specific sites. Hilton et al. (2015) presented that programmable DNA-binding proteins can be fused with p300 domain. These results support targeted acetylation as causal mechanism of *trans*-activation and present a strong tool for gene regulation manipulation. It is noteworthy that effector domains directly catalyzing repressive DNA methylation or histone alterations can be fused with DBDs for making epigenetic silencing proteins. Artificial ZFNs joined with DNMT3a catalyze methylation of DNA and repress transcription from endogenous promoters (Rivenbark et al., 2012; Li et al., 2015).

In the absence of capability to amend the marks at exact sites, researchers are unable to conclude whether they produce biological changes or not. As a result, for plants, system tools need extraordinary refinement for absolute results. In particular case of plant epigenetics, epigenome may be taken as the best mode for controlling activity of gene. In this way, we have to adjust plant epigenome not the plant genome itself. epiCas9 with ability to install/remove unambiguous epigenetic script at specific loci would serve as an additional stage in query of underlying effects of epigenetic amendments in determining the regulatory set-ups of genome. Obviously, the prospective for off-target doings and crosstalk between endogenous epigenetic complexes and effector domains need careful and competent characterization. One way out might be the harnessing of prokaryotic epigenetic enzymes for development of orthogonal epigenetic regulatory mechanisms that can reduce crosstalk with endogenous proteins.

### Model Crop Plants

Undoubtedly, crop biologists are striving hard to engineer resistance against diseases, enhancing tolerance to low precipitation or survival under degraded rhizosphere by introducing advantageous genes taken from other varieties of similar species. But no one can deny the fact that adoption of conventional breeding to move traits may take several years and precision is still questionable. Despite the earlier met failures in establishing plant gene targeting technology (Puchta, 1999; Hsu et al., 2014), at the moment single genes are on target of CRISPR-Cas9 system rather than whole genome (Puchta and Fauser, 2013). Being RNA-guided DNA endonuclease, Cas9 can target to explicit genome sequence for making complex with the help of discretely engineered guided RNA (Wang et al., 2011; Quetier, 2015). Generally, CRISPR-Cas9 is highly adaptable for editing of plant genome (Charpentier and Doudna, 2013; Schaeffer and Nakata, 2015) but especially appropriate for genome editing of monocotyledons, e.g., rice due to high genomic GC content (Miao et al., 2013). With special reference to economically valuable plants, i.e., crops and ornamentals, this technique offers an extraordinary and pragmatic system to produce novel phenotypes.

By applying synthetic nucleases, we are able to introduce delicate changes in genome of crop plants by initiating natural repairing pathways, e.g., NHEJ can induct mutations (Puchta and Fauser, 2014). Of special concern is the point that foreign genes can be introduced through NHEJ or HR anywhere in point of interest activated by any DSB. Definitely this is the beginning. Much more is waiting ahead.

After successful demonstration as a genome editor in widely used lab plants *Arabidopsis thaliana* and *Nicotiana benthamiana*, CRISPR- Cas9 has been tested in different crops, e.g., rice, wheat, sorghum, soybeans, tomatoes, and oranges. In agriculture, CRISPR-Cas9 is presently being employed to knock-out unwanted genes from crops to promote preferable traits. For example, Chinese researchers developed wheat line resistant to powdery mildew. Genome editing may escort to a few surprising developments in agriculture. Different allergy causing proteins have been detected in peanuts (Hourihane et al., 1997; Skolnick et al., 2001). Getting rid of these proteins is not easy. But new technology may likely to offer allergy-free peanuts. CRISPR-Cas9 technique advocates important changes in plant genome within our access. Gene editing can help in overcoming a hurdle that is polyploid plants showing duplicate genome copies, i.e., Wheat. Successful editing of wheat genome in China demonstrates that CRISPR-Cas9 is definitely “multiplexed” with enormous ability to affect all gene copies or to target several genes at the same time. Certainly, any redundant natural sequence may positively be removed from plant genome by adopting this technique and designing model plants. In different chromosomes, induction of two DSBs may facilitate chromosome arms exchange (Lee et al., 2012). This supports origin of variations for survival by means of available variety of raw material (Puchta and Fauser, 2013). Genome engineering with the help of DSB is now being combined with site-specific recombinase technology in plants of economic importance especially, i.e., rice (Wang et al., 2011).

With simultaneous modification of multiple traits, the CRISPR-Cas9 system would provide highly competent method to pyramid breeding (Bortesi and Fischer, 2015). Gene knockouts mediated by NHEJ are the most direct function of CRISPR-Cas9. Negative regulators of plant disease resistance and grain development can be amended for increasing yield and granting resistance to the host plant against targeted pathogens (Song et al., 2016). Other gene editing strategies, for example regulation of gene expression and epigenetic modulation, can also be adopted for increasing agricultural benefits. Moreover, CRISPR-Cas9 offers unconventional approaches, i.e., Cas9 protein-sgRNA ribonucleoproteins, to deliver target genes into crops with no transgenic footprint. By this way we can circumvent the routine regulations on GMOs (Woo et al., 2015).

Setting up new dimensions in plant science, it tempts to speculate that chromosome engineering and plant genome construction via CRISPR-Cas9 technology is no more a dream. Improvements in nutritional values would be welcome in many crop species and some of them can be approached sensibly through genome editing (Baltes and Voytas, 2015). Extraordinarily, in few plant species, knockout mutants of homozygous nature can be created in distinct generation. Jointly with rest of sequence-specific nucleases, CRISPR-Cas9 is really a game changer skill aimed at revolutionary transformation in plant sciences.

### Highly Efficient Plant Cell System

Besides application of classical methods of genetics and breeding for improvements, genome editing through Cas9 has accelerated the efforts for generating the best transgenic models and augment scientific research (Sander and Joung, 2014). Today, mutations in diseased plant populations have been focused. It is considered that CRISPR-based genome editing would be helpful in determining the underlying works of exact genetic abnormalities instead of reliance upon crop disease models. By following the same theme, technique has been applied for developing transgenic animal models few years ago (Niu et al., 2014). iPS cell disease model were engineered with definite mutations corrected or introduced with gene correction in animals (Schwank et al., 2013). The Cas9 genome editing efficiency has made it possible to modify several targets simultaneously, thus facilitates impartial genome-wide functional screens to categorize genes performing central role in development of desired phenotype. Lentivirally delivered sgRNAs directed against all genes can be used to agitate a large number of genomic elements simultaneously (Hsu et al., 2014).

For producing model plant cell systems with high efficiency, constant CRISPR-Cas9expression can be applied for mutants with super efficiency (Xing et al., 2014). These multipurpose systems can be efficiently employed for achieving objectives including production of medicinal and industrial compounds, developing resistance against abiotic as well as biotic stresses (Baltes and Voytas, 2015). Cas9 can simply be launched into the targeted cells by using transitory plasmid transfection having Cas9 and the suitable sgRNA. Like humans, Genome wide association study (GWAS) (Hsu et al., 2014) may appear useful in identifying haplotypes showing positive association with disease risk. One main aim of this technique is to design and create a cell, mean to engineer a cell of desired characteristics with no unwanted component and with ability to divide and pass triplet code ahead. Although likely plant cell is still a dream but methodologies for generating such cells have be employed. For example, CRISPR technique was used to get rid of kilobases of rice DNA that were unnecessary for plant growth. General speaking, such deletions of bases or gene knockouts will be of paramount significance for advancement. But, a strong understanding of needed or unwanted genetic components would chiefly facilitate this series.

The capability of CRISPR-Cas9 system to stack many genes is also feasible with probable application in approaches like metabolic engineering and molecular farming (Mohan, 2016). Recently it is revealed that benzylisoquinoline alkaloid (BIA) production in poppy can be modulated by modifying the particular genes expression in the BIA pathway. Transient over-expression or TRV-mediated gene silencing studies in opium poppy demonstrated that the quantity of BIA biosynthesis could be influenced in a tissue-specific mode (Hosseini et al., 2011; Desgagné-Penix and Facchini, 2012). The over-expression and the silencing of 7OMT and 4′OMT2 genes (R, S)-reticuline 7-*O*-methyltransferase, 3′-hydroxyl-*N*-methylcoclaurine 4′-*O*-methyltransferase) collectively proved their regulatory functions in BIA synthesis in different plant tissues. The previous strategies influenced gene expression that resulted in major reduction in gene expression but never abolished gene function (Alagoz et al., 2016). Therefore, the application CRISPR/Cas9 for knocking out such genes can help to address the challenges and increment our understanding plant cell systems.

By using this technique, we can examine the effect of single gene alternative or experiment the consequence of sole gene maneuvering on isogenic background by editing cells and then differentiating into cell of interest. Such advancements are expected to facilitate gainful, large-scale and less time consuming *in vivo* mutagenesis studies for avoiding perplexing off-target mutagenesis. Furthermore, Cas9 can be strap up for straight alteration of somatic tissues, precluding the requirement for embryonic exploitation and gene therapy. Although challenges to be addressed are many yet the CRISPR-Cas9 system will certainly evolve into a comprehensive strategy for biotechnology and précised crop breeding in near future. We are hopeful that with the passage of time remarkable advances would be possible in terms of genome editing in plants to produce improved plant systems with desirable traits.

## Concluding Remarks and Future Perspectives

Since beginning of this decade, genome editing systems have been adopted to achieve a wide range of modifications, from subtle nucleotide alterations within host genes to the deletion of megabases in DNA (Chen et al., 2016). Together with well-defined and programmable DNA components, plant genome engineering has great potential to facilitate ambitious projects in plant biology. The availability of the CRISPR-Cas9 technology will assist the growing genomics and systems biology data to be exploited very comprehensively by accelerating discovery of genes and related traits development among plant species (**Tables 5** and **6**). Most of the CRISPR-Cas9 related information is currently obtained from research conducted in mammals. Apparently it is assessed that several of these findings can be universal yet it is imperative to execute analogous studies in plants to make sure that system characteristics are translatable to diverse species. This positively applies to extensive applications like orthogonal gene targeting that have yet to be experienced in plant systems (Bortesi and Fischer, 2015).

**Table 6:** Specific commercial products and services available to the researchers to implement CRISPR technology.

Commercial sources	Products and services			
*GeneCopoeia*	Genome-wide sgRNA clones	HDR donor cloning vectors and custom HDR donor construction.	Cas9 stable cell lines.	Insertion/deletion detection system
*Sigma–Aldrich*	CRISPR Selection Too	Paired nickases	Codon-optimized Cas9	Transfection-grade CRISPR plasmid with a guide RNA
*Bio Labs England*	Q5 Hot Start High-Fidelity 2X Master Mix, NEBuilder HiFi DNA Assembly Master Mix	Q5 Site-Directed Mutagenesis Kit (with competent cells) and Q5 Site-Directed Mutagenesis Kit (Without Competent Cells)	EnGe Cas9 Nuclease	EnGen Mutation Detection Kit
*Integrated DNA technologies (IDT)*	Human HPRT PCR Primer Mix, Mouse HPRT PCR Primer Mix, Nuclease Free Duplex Buffer	S.p. Cas9 Expression Plasmid	S.p. Cas9 Nuclease 3NLS (100, 500 μg)	CRISPR Negative Control crRNA, CRISPR Positive Control crRNA
*DNA 2.0*	Nickase Ninja All-in-One construct expressing specific dual gRNAs	Electra Cloning Kit		
*Cyagen*	ROSA26 large fragment knockin			
*ORiGene*	CRISPR/Cas starter kit (HA tagging human HSP60 at C-terminus).	pCas-Guide-Nickase (D10A), pT7-Cas9-Nickase (D10A)	pCas-Guide Cloning Kit,	pCas-Guide-scramble (also available as negative control)
*System Biosciences*	Multiplex gRNA Cloning Kit Create CRISPR/Cas9 constructs with multiple gRNAs simultaneously for better genome editing			
*Eurofins Genomics*	Cloning Oligos	Indel Detection by Amplicon Analysis	Custom Sequencing (Check the sequence of your CRISPR plasmid or genomic target sequence)	

*This table is provided only to facilitate researchers and R&D. It does not involve any commercial affiliation, marketing or financial purpose.*

Future research for improving this technology will include optimization of sgRNA scaffold, which is vital for the TE due to its binding affinity for Cas9 (Jinek et al., 2014). Researchers working in polyploid crops like sugarcane, wheat need information about variation of sequence among diverse allelic forms to design precise gRNAs (Mohan, 2016). Moreover, direct engineering of these Cas9 proteins from diverse bacterial types should tender a path toward PAM independence and producing more competent Cas9 proteins. The extent of off-target mutations and differences in cleavage efficiency need to be evaluated more precisely. Another conspicuous challenge ahead is absence of high throughput screening methods to recognize transgenic plants with edited gene events. In parallel with other studies, capability of CRSIPR-Cas9 system to generate and test multiple gRNAs and availability of next-generation sequencing (NGS) technologies will grant adequate data for the comparison of this system in diversity of plant species and cell types. Keeping in view the number of researchers engaged in CRISPR-Cas9 and velocity of this technique development, additional increments in our understanding and control of the system are expected to come swiftly, promisingly guiding to the devise a new batch of genome editing tools.

## Author Contributions

AN has collected research data and compiled manuscript. MA has made all figures and tables. SH has evaluated this manuscript and corrected mistakes.

## Conflict of Interest Statement

The authors declare that the research was conducted in the absence of any commercial or financial relationships that could be construed as a potential conflict of interest.

## References

[B1] Abdallah N. A. Prakash C. S. McHughen A. G. (2015). Genome editing for crop improvement: Challenges and opportunities. *GM Crops Food* 6 183–205. 10.1080/21645698.2015.1129937 26930114 PMC5033222

[B2] Ainley W. M. Sastry-Dent L. Welter M. E. Murray M. G. Zeitler B. Amora R. (2013). Trait stacking via targeted genome editing. *Plant Biotechnol. J.* 11 1126–1134. 10.1111/pbi.12107 23953646

[B3] Alagoz Y. Gurkok T. Zhang B. Unver T. (2016). Manipulating the biosynthesis of bioactive compound alkaloids for next-generation metabolic engineering in opium poppy using CRISPR-Cas 9 genome editing technology. *Sci. Rep.* 6:30910 10.1038/srep30910 PMC4971470 27483984

[B4] Baltes N. J. Gil-Humanes J. Cermak T. Atkins P. A. Voytas D. F. (2014). DNA replicons for plant genome engineering. *Plant Cell* 26 151–163. 10.1105/tpc.113.119792 24443519 PMC3963565

[B5] Baltes N. J. Voytas D. F. (2015). Enabling plant synthetic biology through genome engineering. *Trends Biotechnol.* 33 120–131. 10.1016/j.tibtech.2014.11.008 25496918

[B6] Bolotin A. Quinquis B. Sorokin A. Ehrlich S. D. (2005). Clustered regularly interspaced short palindrome repeats (CRISPRs) have spacers of extra chromosomal origin. *Microbiology* 151 2551–2561. 10.1099/mic.0.28048-0 16079334

[B7] Bortesi L. Fischer R. (2015). The CRISPR system for plant genome editing and beyond. *Biotechnol. Adv.* 33 41–52. 10.1016/j.biotechadv.2014.12.006 25536441

[B8] Char S. N. Unger-Wallace E. Frame B. Briggs S. A. Main M. Spalding M. H. (2015). Heritable site-specific mutagenesis using TALENs in maize. *Plant Biotechnol. J.* 13 1002–1010. 25644697 10.1111/pbi.12344

[B9] Charpentier E. Doudna J. A. (2013). Rewriting a genome. *Nature* 495 50–51. 10.1038/495050a 23467164

[B10] Chen B. Gilbert L. A. Cimini B. A. Schnitzbauer J. Zhang W. Li G. W. (2013). Dynamic imaging of genomic loci in living human cells by an optimized CRISPR/Cas system. *Cell* 155 1479–1491. 10.1016/j.cell.2013.12.001 24360272 PMC3918502

[B11] Chen B. Guan J. Huang B. (2015). Imaging specific genomic DNA in living cells. *Annu. Rev. Biophys.* 45 1–23. 10.1146/annurev-biophys-062215-010830 PMC5053920 27145877

[B12] Chen B. Hu J. Almeida R. Liu H. Balakrishnan S. Covill-Cooke C. (2016). Expanding the CRISPR imaging toolset with *Staphylococcus aureus* Cas9 for simultaneous imaging of multiple genomic loci. *Nucleic Acids Res.* 44:e75 10.1093/nar/gkv153.3 PMC4856973 26740581

[B13] Cho S. W. Kim S. Kim J. M. Kim J. S. (2013). Targeted genome engineering in human cells with the Cas9 RNA-guided endonuclease. *Nat. Biotechnol.* 31 230–232. 10.1038/nbt.2507 23360966

[B14] Cong L. Ran F. A. Cox D. Lin S. Barretto R. Habib N. (2013). Multiplex genome engineering using CRISPR/Cas systems. *Science* 339 819–823. 10.1126/science.1231143 23287718 PMC3795411

[B15] Cook C. Martin L. Bastow R. (2014). Opportunities in plant synthetic biology. *J. Exp. Bot.* 65 1921–1926. 10.1093/jxb/eru013 24502956

[B16] Deltcheva E. Chylinski K. Sharma C. M. Gonzales K. Chao Y. Pirzada Z. A. (2011). CRISPR RNA maturation by trans-encoded small RNA and host factor RNase III. *Nature.* 471 602–607. 10.1038/nature09886 21455174 PMC3070239

[B17] Desgagné-Penix I. Facchini P. J. (2012). Systematic silencing of benzylisoquinoline alkaloid biosynthetic genes reveals the major route to papaverine in opium poppy. *Plant J.* 72 331–344. 10.1111/j.1365-313X.2012.05084.x 22725256

[B18] Ding Q. Regan S. N. Xia Y. Oostrom L. A. Cowan C. A. Musunuru K. (2013). Enhanced efficiency of human pluripotent stem cell genome editing through replacing TALENs with CRISPRs. *Cell Stem Cell* 12 393–394. 10.1016/j.stem.2013.03.006 23561441 PMC3925309

[B19] D’Halluin K. Vanderstraeten C. Van Hulle J. Rosolowska J. Van Den Brande I. Ennewaert A. (2013). Targeted molecular trait stacking in cotton through targeted double-strand break induction. *Plant Biotechnol. J.* 11 933–941. 10.1111/pbi.12085 23777410 PMC4272417

[B20] Fauser F. Schiml S. Puchta H. (2014). Both CRISPR/Cas-based nucleases and nickases can be used efficiently for genome engineering in *Arabidopsis thaliana*. *Plant J.* 79 348–359. 10.1111/tpj.12554 24836556

[B21] Feng Z. Mao Y. Xu N. Zhang B. Wei P. Yang D. L. (2014). Multigeneration analysis reveals the inheritance, specificity and patterns of CRISPR/Cas-induced gene modifications in *Arabidopsis*. *Proc. Natl. Acad. Sci. U S A.* 111 4632–4637. 24550464 10.1073/pnas.1400822111 PMC3970504

[B22] Feng Z. Zhang B. Ding W. Liu X. Yang L. Y. Wei P. (2013). Efficient genome editing in plants using a CRISPR/Cas system. *Cell Res.* 23 1229–1232. 10.1038/cr.2013.114 23958582 PMC3790235

[B23] Fu Y. Foden J. A. Khayter C. Maeder M. L. Reyon D. Joung J. K. (2013). High-frequency off-target mutagenesis induced by CRISPR-Cas nucleases in human cells. *Nat. Biotechnol.* 31 822–826. 10.1038/nbt.2623 23792628 PMC3773023

[B24] Fu Y. Sander J. D. Reyon D. Cascio V. M. Joung J. K. (2014). Improving CRISPR-Cas nuclease specificity using truncated guide RNAs. *Nat. Biotechnol.* 32 279–284. 10.1038/nbt.2808 24463574 PMC3988262

[B25] Gao H. Smith J. Yang M. Jones S. Djukanovic V. Nicholson M. G. (2010). Heritable targeted mutagenesis in maize using a designed endonuclease. *Plant J.* 61 176–187. 10.1111/j.1365-313X.2009.04041.x 19811621

[B26] Gasiunas G. Barrangou R. Horvath P. Siksnys V. (2012). Cas9-crRNA ribonucleoprotein complex mediates specific DNA cleavage for adaptive immunity in bacteria. *Proc. Natl. Acad. Sci. U.S.A.* 109 E2579–E2586. 10.1073/pnas.1208507109 22949671 PMC3465414

[B27] Gregory D. J. Zhang Y. Kobzik L. Fedulov A. V. (2013). Specific transcriptional enhancement of inducible nitric oxide synthase by targeted promoter demethylation. *Epigenet* 8 1205–1212. 10.4161/epi.26267 PMC5845820 24008769

[B28] Grewal S. I. Jia S. (2007). Heterochromatin revisited. *Nat. Rev. Genet.* 8 35–46. 10.1038/nrg2008 17173056

[B29] Griffith J. D. Comeau L. Rosenfield S. Stansel R. M. Bianchi A. Moss H. (1999). Mammalian telomeres end in a large duplex loop. *Cell* 97 503–514. 10.1016/S0092-8674(00)80760-6 10338214

[B30] Haft D. H. Selengut J. Mongodin E. F. Nelson K. E. (2005). A guild of 45 CRISPR-associated (Cas) protein families and multiple CRISPR/Cas subtypes exist in prokaryotic genomes. *PLoS Comput. Biol.* 1:e60 10.1371/journal.pcbi.0010060.eor PMC1282333 16292354

[B31] Hale C. R. Zhao P. Olson S. Duff M. O. Graveley B. R. Wells L. (2009). RNA-guided RNA cleavage by a CRISPR RNA-Cas protein complex. *Cell* 139 945–956. 10.1016/j.cell.2009.07.040 19945378 PMC2951265

[B32] Hilton I. B. D’Ippolito A. M. Vockley C. M. Thakore P. I. Crawford G. E. Reddy T. E. (2015). Epigenome editing by a CRISPR-Cas9-based acetyltransferase activates genes from promoters and enhancers. *Nat. Biotechnol.* 33 510–517. 10.1038/nbt.3199 25849900 PMC4430400

[B33] Hosseini B. Shahriari-Ahmadi F. Hashemi H. Marashi M. H. Mohseniazar M. Farokhzad A. (2011). Transient expression of cor gene in *Papaver somniferum*. *Bioimpacts* 1 229–235. 23678433 10.5681/bi.2011.033 PMC3648975

[B34] Hourihane J. O. Kilburn S. A. Dean P. Warner J. O. (1997). Clinical characteristics of peanut allergy. *Clin. Exp. Allergy* 27 634–639. 9208183

[B35] Hsu P. D. Lander E. S. Zhang F. (2014). Development and applications of CRISPRfor genome engineering. *Cell* 157 1262–1278. 10.1016/j.cell.2014.05.010 24906146 PMC4343198

[B36] Hsu P. D. Scott D. A. Weinstein J. A. Ran F. A. Konermann S. Agarwala V. (2013). DNA targeting specificity of RNA-guided Cas9 nucleases. *Nat. Biotechnol.* 31 827–832. 10.1038/nbt.2647 23873081 PMC3969858

[B37] Iida S. Terada R. (2005). Modification of endogenous natural genes by gene targeting in rice and other higher plants. *Plant Mol. Biol.* 59 205–219. 10.1007/s11103-005-2162-x 16217613

[B38] Ishino Y. Shinagawa H. Makino K. Amemura M. Nakata A. (1987). Nucleotide sequence of the iap gene, responsible for alkaline phosphatase isozyme conversion in *Escherichia coli* and identification of the gene product. *J. Bacteriol.* 169 5429–5433. 10.1128/jb.169.12.5429-5433.1987 3316184 PMC213968

[B39] Jiang H. Wang N. (2014). Targeted genome editing of sweet orange using Cas9/sgRNA. *PLoS ONE* 9:e93806. 10.1371/journal.pone.0093806 PMC3977896 24710347

[B40] Jiang W. Zhou H. Bi H. Fromm M. Yang B. Weeks D. P. (2013). Demonstration of CRISPR/sgRNA-mediated targeted gene modification in *Arabidopsis*, tobacco, sorghum and rice. *Nucleic Acids Res.* 41:e188 10.1093/nar/gkt780 PMC3814374 23999092

[B41] Jiang W. Z. Yang B. Weeks D. P. (2014). Efficient CRISPR-mediated gene editing in *Arabidopsis thaliana* and inheritance of modified genes in the T2 and T3 generations. *PLoS ONE* 9:e99225 10.1371/journal.pone.0099225 PMC4053344 24918588

[B42] Jinek M. Chylinski K. Fonfara I. Hauer M. Doudna J. A. Charpentier E. (2012). A programmable dual-RNA-guided DNA endonuclease in adaptive bacterial immunity. *Science* 337 816–821. 10.1126/science.1225829 22745249 PMC6286148

[B43] Jinek M. Jiang F. Taylor D. W. Sternberg S. H. Kaya E. Ma E. (2014). Structures of Cas9 endonucleases reveal RNA-mediated conformational activation. *Science* 343:1247997 10.1126/science.1247997 PMC4184034 24505130

[B44] Kalhor R. Tjong H. Jayathilaka N. Alber F. Chen L. (2012). Genome architectures revealed by tethered chromosome conformation capture and population-based modeling. *Nat. Biotechnol.* 30 90–98. 10.1038/nbt.2057 PMC3782096 22198700

[B45] Kanchiswamy C. N. Maffei M. Malnoy M. Velasco R. Kim J. S. (2016). Fine-tuning next-generation genome editing tools. *Trends Biotechnol.* 34 562–574. 10.1016/j.tibtech.2016.03.007 27167723

[B46] Kochevenko A. Willmitzer L. (2003). Chimeric RNA/DNA oligonucleotide based site-specific modification of the tobacco acetolactatesyntase gene. *Plant Physiol.* 132 174–184. 10.1104/pp.102.016857 12746523 PMC166963

[B47] Konermann S. Brigham M. D. Trevino A. E. Joung J. Abudayyeh O. O. Barcena C. (2015). Genome-scale transcriptional activation by an engineered CRISPR-Cas9 complex. *Nature* 515 83–88. 10.1038/nature14136 PMC4420636 25494202

[B48] Kumar V. Jain M. (2014). The CRISPR–Cas system for plant genome editing: advances and opportunities. *J. Exp. Bot.* 66 47–57. 10.1093/jxb/eru429 25371501

[B49] Ladford H. (2016). CRISPR: gene editing is just the beginning. *Nature* 531 156–159. 10.1038/531156a 26961639

[B50] Lee H. J. Kweon J. Kim E. Kim S. Kim J. S. (2012). Targeted chromosomal duplications and inversions in the human genome using zinc finger nucleases. *Genome Res.* 22 539–548. 10.1101/gr.129635.111 22183967 PMC3290789

[B51] Li J. F. Norville J. E. Aach J. McCormack M. Zhang D. Bush J. (2013). Multiplex and homologous recombination-mediated genome editing in *Arabidopsis* and *Nicotiana benthamiana* using guide RNA and Cas9. *Nat. Biotechnol.* 31 688–691. 10.1038/nbt.2654 23929339 PMC4078740

[B52] Li K. Pang J. Cheng H. Liu W. P. Di J. M. Xiao H. J. (2015). Manipulation of prostate cancer metastasis by locus-specific modification of the CRMP4 promoter region using chimeric TALE DNA methyltransferase and demethylase. *Oncotarget* 6 10030–10044. 10.18632/oncotarget.3192 25888628 PMC4496338

[B53] Ma Y. Zhang L. Huang X. (2014). Genome modification by CRISPR. *FEBS J.* 281 5186–5193. 10.1111/febs.13110 25315507

[B54] Maeder M. L. Angstman J. F. Richardson M. E. Linder S. J. Cascio V. M. Tsai S. Q. (2013a). Targeted DNA demethylation and activation of endogenous genes usingprogrammable TALE-TET1 fusion proteins. *Nat. Biotechnol.* 31 1137–1142. 10.1038/nbt.2726 24108092 PMC3858462

[B55] Maeder M. L. Linder S. J. Cascio V. M. Fu Y. Ho Q. H. Joung J. K. (2013b). CRISPR RNA-guided activation of endogenous human genes. *Nat. Methods* 10 977–979. 10.1038/nmeth.2598 23892898 PMC3794058

[B56] Maeder M. L. Thibodeau-Beganny S. Osiak A. Wright D. A. Anthony R. M. Eichtinger M. (2008). Rapid “open-source” engineering of customized zinc-finger nucleases for highly efficient gene modification. *Mol. Cell* 31 294–301. 10.1016/j.molcel.2008.06.016 18657511 PMC2535758

[B57] Mak A. N. Bradley P. Cernadas R. A. Bogdanove A. J. Stoddard B. L. (2012). The crystal structure of TAL effector PthXo1 bound to its DNA target. *Science* 335 716–719. 10.1126/science.1216211 22223736 PMC3427646

[B58] Malina A. Katigbak A. Cencic R. Maïga R. I. Robert F. Miura H. (2014). Adapting CRISPR for functional genomics screens. *Methods Enzymol.* 546 193–213. 10.1016/B978-0-12-801185-0.00010-6 25398342

[B59] Mao Y. Zhang H. Xu N. Zhang B. Gou F. Zhu J. K. (2013). Application of the CRISPR-Cas system for efficient genome engineering in plants. *Mol. Plant* 6 2008–2011. 10.1093/mp/sst121 23963532 PMC3916745

[B60] Mendenhall E. M. Williamson K. E. Reyon D. Zou J. Y. Ram O. Joung J. K. (2013). Locus-specific editing of histone modifications at endogenous enhancers. *Nat. Biotechnol.* 31 1133–1136. 10.1038/nbt.2701 24013198 PMC3858395

[B61] Miao J. Guo D. Zhang J. Huang Q. Qin G. Zhang X. (2013). Targeted mutagenesis in rice using CRISPR-Cas system. *Cell Res.* 23 1233–1236. 10.1038/cr.2013.123 23999856 PMC3790239

[B62] Miller J. C. Tan S. Qiao G. Barlow K. A. Wang J. Xia D. F. (2011). A tale nuclease architecture for efficient genome editing. *Nat. Biotechnol.* 29 143–148. 10.1038/nbt.1755 21179091

[B63] Mohan C. (2016). Genome editing in sugarcane: challenges ahead. *Front. Plant Sci.* 7:1542 10.3389/fpls.2016.01542 PMC5061775 27790238

[B64] Nagano T. Lubling Y. Stevens T. J. Schoenfelder S. Yaffe E. Dean W. (2013). Single-cell Hi-C reveals cell-tocell variability in chromosome structure. *Nature* 502 59–64. 10.1038/nature12593 24067610 PMC3869051

[B65] Nekrasov V. Staskawicz B. Weigel D. Jones J. D. Kamoun S. (2013). Targeted mutagenesis in the model plant *Nicotiana benthamiana* using Cas9 RNA-guided endonuclease. *Nat. Biotechnol.* 31 691–693. 10.1038/nbt.2655 23929340

[B66] Neumann H. Neumann-Staubitz P. (2010). Synthetic biology approaches in drug discovery and pharmaceutical biotechnology. *Appl. Microbiol. Biotechnol.* 87 75–86. 10.1007/s00253-010-2578-3 20396881 PMC2872025

[B67] Nemudryi A. A. Valetdinova K. R. Medvedev S. P. Zakian S. M. (2014). TALEN and CRISPR/Cas genome editing systems: tools of discovery. *Acta Nat.* 6 19–40. PMC4207558 25349712

[B68] Niu Y. Shen B. Cui Y. Chen Y. Wang J. Wang L. (2014). Generation of gene-modified cynomolgus monkey via Cas9/RNA-mediated gene targeting in one-cell embryos. *Cell* 156 836–843. 10.1016/j.cell.2014.01.027 24486104

[B69] Oldroyd G. E. Murray J. D. Poole P. S. Downie J. A. (2011). The rules of engagement in the legume–rhizobial symbiosis. *Ann. Rev. Gen.* 45 119–144. 10.1146/annurev-genet-110410-132549 21838550

[B70] Oldroyd G. E. D. Dixon R. (2014). Biotechnological solutions to the nitrogen problem. *Curr. Opin. Biotechnol.* 26 19–24. 10.1016/j.copbio.2013.08.006 24679253

[B71] Perez-Pinera P. Kocak D. D. Vockley C. M. Adler A. F. Kabadi A. M. Polstein L. R. (2013). RNA-guided gene activation by CRISPR-Cas9-based transcription factors. *Nat. Methods* 10 973–976. 10.1038/nmeth.2600 23892895 PMC3911785

[B72] Piatek A. Ali Z. Baazim H. Li L. Abulfaraj A. Al-Shareef S. (2014). RNA-guided transcriptional regulation in planta via synthetic dCas9 –based transcription factors. *Plant Biotechnol. J.* 13 578–589. 10.1111/pbi.12284 25400128

[B73] Puchta H. (1999). Double-strand break-induced recombination between ectopic homologous sequences in somatic plant cells. *Genetics* 152 1173–1181. 10388832 10.1093/genetics/152.3.1173 PMC1460648

[B74] Puchta H. Fauser F. (2013). Gene targeting in plants: 25 years later. *Int. J. Dev. Biol.* 57 629–637. 10.1387/ijdb.130194hp 24166445

[B75] Puchta H. Fauser F. (2014). Synthetic nucleases for genome engineering in plants: prospects for a bright future. *Plant J.* 78 727–741. 10.1111/tpj.12338 24112784

[B76] Qi L. S. Larson M. H. Gilbert L. A. Doudna J. A. Weissman J. S. Arkin A. P. (2013). Repurposing CRISPR as an RNA-guided platform for sequence-specific control of gene expression. *Cell* 152 1173–1183. 10.1016/j.cell.2013.02.022 23452860 PMC3664290

[B77] Quetier F. (2015). The CRISPRtechnology:Closer to the ultimate toolkit for targeted genome editing. *Plant Sci.* 242 65–76. 10.1016/j.plantsci.2015.09.003 26566825

[B78] Ran F. A. Hsu P. D. Lin C. Y. Gootenberg J. S. Konermann S. Trevino A. E. (2013). Double nicking by RNA-guided CRISPR Cas9 for enhanced genome editing specificity. *Cell* 154 1380–1389. 10.1016/j.cell.2013.08.021 23992846 PMC3856256

[B79] Rivenbark A. G. Stolzenburg S. Beltran A. S. Yuan X. Rots M. G. Strahl B. D. (2012). Epigenetic reprogramming of cancer cells via targeted DNA methylation. *Epigenet* 7 350–360. 10.4161/epi.19507 PMC3368819 22419067

[B80] Roukos V. Voss T. C. Schmidt C. K. Lee S. Wangsa D. Misteli T. (2013). Spatial dynamics of chromosome translocations in living cells. *Science* 341 660–664. 10.1126/science.1237150 23929981 PMC6324928

[B81] Sander J. D. Joung J. K. (2014). CRISPR-Cas systems for editing, regulating and targeting genomes. *Nat. Biotechnol.* 32 347–355. 10.1038/nbt.2842 24584096 PMC4022601

[B82] Sarrion-Perdigones A. Vazquez-Vilar M. Palaci J. Castelijns B. Forment J. Ziarsolo P. (2013). GoldenBraid 2.0: a comprehensive DNA assembly framework for plant synthetic biology. *Plant Physiol.* 162 1618–1631. 10.1104/pp.113.217661 23669743 PMC3707536

[B83] Schaeffer S. M. Nakata P. A. (2015). CRISPR-mediated genome editing and gene replacement in plants: transitioning from lab to field. *Plant Sci.* 240 130–142. 10.1016/j.plantsci.2015.09.011 26475194

[B84] Schiml S. Fauser F. Puchta H. (2014). The CRISPR/Cas system can be used as nuclease for in planta gene targeting and as paired nickases for directed mutagenesis in *Arabidopsis* resulting in heritable progeny. *Plant J.* 80 1139–1150. 10.1111/tpj.12704 25327456

[B85] Schwank G. Koo B. K. Sasselli V. Dekkers J. F. Heo I. Demircan T. (2013). Functional repair of CFTR by CRISPR in intestinal stem cell organoids of cystic fibrosis patients. *Cell Stem Cell* 13 653–658. 10.1016/j.stem.2013.11.002 24315439

[B86] Shan Q. Wang Y. Chen K. Liang Z. Li J. Zhang Y. (2013a). Rapid and efficient gene modification in rice and *Brachypodium* using TALENs. *Mol. Plant* 6 1365–1368. 10.1093/mp/sss162 23288864 PMC3968307

[B87] Shan Q. Wang Y. Li J. (2013b). Targeted genome modification of crop plants using a CRISPR–Cas system. *Nat. Biotechnol.* 31 686–688. 10.1038/nbt.2650 23929338

[B88] Shan Q. Wang Y. Li J. Zhang Y. Chen K. Liang Z. (2013c). Targeted genome modification of crop plants using the CRISPR–Cassystem.N*at*. *Biotechnology* 31 686–688. 10.1038/nbt.2650 23929338

[B89] Shan Q. Wang Y. Li J. Gao C. (2014). Genome editing in rice and wheat using CRISPR/Cas system. *Nat. Protoc.* 9 2395–2410. 10.1038/nprot.2014.157 25232936

[B90] Shao S. Zhang W. Hu H. Xue B. Qin J. (2016). Long-term dual-color tracking of genomic loci by modified sgRNAs of the CRISPR/Cas9 system. *Nucleic Acids Res.* 44 e86. 10.1093/nar/gkw066 PMC4872083 26850639

[B91] Skolnick H. S. Conover-Walker M. K. Koerner C. B. Sampson H. A. Burks W. Wood R. A. (2001). The natural history of peanut allergy. *J. Allergy Clin. Immunol.* 107 367–374. 10.1067/mai.2001.112129 11174206

[B92] Song G. Jia M. Chen K. Kong X. Khattak B. Xie C. (2016). CRISPR: a powerful tool for crop genome editing. *Crop J.* 4 75–82. 10.1016/j.cj.2015.12.002

[B93] Sozzani R. Cui H. Moreno-Risueno M. A. Busch W. Van Norman J. M. Vernoux T. (2010). Spatiotemporal regulation of cell-cycle genes by short root links patterning and growth. *Nature* 466 128–132. 10.1038/nature09143 20596025 PMC2967763

[B94] Sprink T. Metje J. Hartung F. (2015). Plant genome editing by novel tools: TALEN and other sequence specific nucleases. *Curr. Opin. Biotechnol.* 32 47–53. 10.1016/j.copbio.2014.11.010 25448232

[B95] Stasevich T. J. Hayashi-Takanaka Y. Sato Y. Maehara K. Ohkawa Y. Sakata-Sogawa K. (2014). Regulation of RNA polymerase II activation by histone acetylation in single living cells. *Nature.* 516 272–275. 10.1038/nature13714 25252976

[B96] Sternberg S. H. Redding S. Jinek M. Greene E. C. Doudna J. A. (2014). DNA interrogation by the CRISPR RNA-guided endonuclease Cas9. *Nature.* 507 62–67. 10.1038/nature13011 24476820 PMC4106473

[B97] Sugano S. S. Shirakawa M. Takagi J. Matsuda Y. Shimada T. Hara-Nishimura I. (2014). CRISPR/Cas9 mediated targeted mutagenesis in the liverwort *Marchantia polymorpha* L. *Plant Cell Physiol.* 55 475–481. 10.1093/pcp/pcu014 24443494

[B98] Temme K. Zhao D. Voigtb C. A. (2012). Refactoring the nitrogen fixation gene cluster from Klebsiellaoxytoca. *Proc. Natl. Acad. Sci. U.S.A.* 109 7085–7090. 22509035 10.1073/pnas.1120788109 PMC3345007

[B99] Thakore P. I. Black J. B. Hilton I. B. Gersbach C. A. (2016). Editing the epigenome: technologies for programmable transcription and epigenetic modulation. *Nat. Methods* 12 127–137. 10.1038/nmeth.3733 PMC4922638 26820547

[B100] Upadhyay S. K. Kumar J. Alok A. Tuli R. (2013). RNA guided genome editing for target gene mutations in wheat. *G3 (Bethesda)* 3 2233–2238. 10.1534/g3.113.008847 24122057 PMC3852385

[B101] Vazquez-Vilar M. Bernabe-Orts J. M. Fernandez-Del-Carmen A. Ziarsolo P. Blanca J. Granell A. (2016). A modular toolbox for gRNA-Cas9 genome engineering in plants based on the golden braid standard. *Plant Methods* 12:10 10.1186/s13007-016-0101-2 PMC4736081 26839579

[B102] Wang H. Yang H. Shivalila C. S. Dawlaty M. M. Cheng A. W. Zhang F. (2013). One-step generation of mice carrying mutations in multiple genes by CRISPR/Cas-mediated genome engineering. *Cell.* 153 910–918. 10.1016/j.cell.2013.04.025 23643243 PMC3969854

[B103] Wang Y. Cheng X. Shan Q. Zhang Y. Liu J. Gao C. (2014). Simultaneous editing of three homoeoalleles in hexaploid bread wheat confers heritable resistance to powdery mildew. *Nat. Biotechnol.* 32 947–951. 10.3410/f.718498343.793497666 25038773

[B104] Wang Y. Yau Y. Y. Perkins-Balding D. Thomson J. G. (2011). Recombinase technology: applications and possibilities. *Plant Cell Rep* 30 267–285. 10.1007/s00299-010-0938-1 20972794 PMC3036822

[B105] Wang Z. P. Xing H. L. Dong L. Zhang H. Y. Han C. Y. Wang X. C. (2015). Egg cell-specific promoter-controlled CRISPR/Cas9 efficiently generates homozygous mutants for multiple target genes in *Arabidopsis* in a single generation. *Genom. Biol.* 16:144 10.1186/s13059-015-0715-0 PMC4507317 26193878

[B106] Wendt T. Holm P. B. Starker C. G. Christian M. Voytas D. F. Brinch-Pedersen H. (2013). TAL effector nucleases induce mutations at a pre-selected location in the genome of primary barley transformants. *Plant Mol. Biol.* 83 279–285. 10.1007/s11103-013-0078-4 23689819 PMC7880306

[B107] Wiedenheft B. Sternberg S. H. Doudna J. A. (2012). RNA-guided genetic silencing systems in bacteria and archaea. *Nature* 82 331–338. 10.1038/nature10886 22337052

[B108] Wolt J. D. Wang K. Yang B. (2016). The regulatory status of genome-edited crops. *Plant Biotechnol. J.* 14 510–518. 10.1111/pbi.12444 26251102 PMC5042095

[B109] Woo J. W. J. Kim J. Kwon S. I. I. Corvalán C. Cho S. W. Kim H. (2015). DNA-free genome editing in plants with preassembled CRISPR-Cas9 ribonucleoproteins. *Nat. Biotechnol.* 33 1162–1164. 10.1038/nbt.3389 26479191

[B110] Xie F. Murray J. D. Kim J. Heckmann A. B. Edwards A. Oldroyd G. E. (2012). Legume pectatelyase required for root infection by rhizobia. *Proc. Natl. Acad. Sci. U.S.A.* 109 633–638. 10.1073/pnas.1113992109 22203959 PMC3258600

[B111] Xing H. L. Li D. Zhi-Ping W. Hai-Yan Z. Chun-Yan H. Bing L. (2014). A CRISPR toolkit for multiplex genome editing in plants. *BMC Plant Biol.* 14:327 10.1186/s12870-014—0327-y PMC4262988 25432517

[B112] Xiong J. S. Ding J. Li Y. (2015). Genome-editing technologies and their potential application in horticultural crop breeding. *Hortic. Res* 2:15019 10.1038/hortres.2015.19 PMC4595993 26504570

[B113] Zalatan J. G. Lee M. E. Almeida R. Gilbert L. A. Whitehead E. H. La Russa M. (2015). Engineering complex synthetic transcriptional programs with CRISPR RNA scaffolds. *Cell* 160 339–350. 10.1016/j.cell.2014.11.052 25533786 PMC4297522

[B114] Zhang F. Wen Y. Guo X. (2014). CRISPR for genome editing: progress, implications and challenges. *Hum. Mol. Genet.* 23 40–46. 10.1093/hmg/ddu125 24651067

[B115] Zhang Y. Zhang F. Li X. Baller J. A. Qi Y. Starker C. G. (2013). Transcription activator-like effector nucleases enable efficient plant genome engineering. *Plant Physiol.* 161 20–27. 10.1104/pp.112.205179 23124327 PMC3532252

[B116] Zhou H. Liu B. Weeks D. P. Spalding M. H. Yang B. (2014). Large chromosomal deletions and heritable small genetic changes induced by CRISPR in rice. *Nucleic Acids Res.* 42 10903–10914. 10.1093/nar/gku806 25200087 PMC4176183

